# Photosynthetic and physiological responses of different peony cultivars to high temperature

**DOI:** 10.3389/fpls.2022.969718

**Published:** 2022-10-28

**Authors:** Wen Ji, Erman Hong, Xia Chen, Zhijun Li, Bangyu Lin, Xuanze Xia, Tianyao Li, Xinzhang Song, Songheng Jin, Xiangtao Zhu

**Affiliations:** ^1^ State Key Laboratory of Subtropical Silviculture, Zhejiang A&F University, Hangzhou, China; ^2^ College of Jiyang, Zhejiang A&F University, Zhuji, China

**Keywords:** high temperature, peony, fluorescence transient, photosynthetic, JIP-test

## Abstract

In order to investigate the causes of the differences in heat tolerance (‘Lu He Hong’ and ‘Zhi Hong’), we studied the physiological changes, photosynthetic properties and regulatory mechanism of the two peony cultivars at high temperature. The results showed that the physiological changed of different peony cultivars varied significantly under high temperature stress. With the extension of high temperature stress time, MDA content of 'Lu He Hong' increased,while 'Zhi Hong' rised first and then decreased, SOD activity of 'Lu He Hong' rised first and then decreased, that of 'Zhi Hong' kept rising, POD activity of 'Lu He Hong' kept decreasing, while 'Zhi Hong' rised. The photosynthetic instrument records the change of peony photosynthesis parameters at high temperature; the chlorophyll A (Chla) fluorescence transient is recorded using the plant efficiency analyzer (PEA), analyzed according to the JIP test (O-J-I-P fluorescence transient analysis), and several parameters were derived to explain the photosynthetic efficiency difference between different peony cultivars. The tested cultivars responded differently to the survey conditions, and the PCA analysis showed that the ‘Zhi Hong’ was more well tolerated and showed better thermal stability of the PSII. The reduced efficiency of the ‘Lu He Hong’ PSII antenna leads to higher heat dissipation values to increase the light energy absorbed by unit reaction center (ABS/RC), the energy captured by unit reaction center (TR_0_/RC), and the energy dissipated by unit reaction center (DI_0_/RC), which significantly leads to its lower total photosynthetic performance (PI_total_). The light capture complex of the variety ‘Zhi Hong’ has high connectivity with its reaction center, less damage to OEC activity, and better stability of the PSII system. The results show that ‘Zhi Hong’ improves heat resistance by stabilizing the cell membrane, a strong antioxidant system, as well as a more stable photosynthetic system. The results of this study provide a theoretical basis for the screening of heat-resistant peonies suitable for cultivation in Jiangnan area and for the selection and breeding of heat-resistant cultivars.

## Introduction

In recent years, with frequent occurrence of extreme weather, global climate change, mainly characterized by temperature rise, has become the focus of worldwide concern (Yu, 2019). High temperature is an important factor affecting crop productivity and plant growth in many regions of the world (Lesk et al., 2016). High temperature stress can adversely affect seed germination and seed viability, leading to thermal damage or death of seeds (Bita and Gerats, 2013). High temperature lead to various plant physiological changes such as scorched leaves and stems, leaf abscission and senescence, stem and root growth inhibition or reduced numbers, pollen tube growth and pollen sterility, and fruit damage leading to catastrophic loss of crop yield resulting in severe loss of productivity (Hemantaranjan et al., 2014). For example, high temperature can have a significant negative impact on the reproductive phenological stages of flowering, silk formation and seed filling in maize (Tiwari and Yadav, 2019), common bean plants are subjected to high temperature stress that restricts pollen tube growth and reduces pollen viability, resulting in low pod and fruit set rates (Gross and Kigel, 1994), rice tasseling and flowering above 35°C do not proceed normally and fertilization rates are significantly reduced (Morita et al., 2005). To counteract the damage caused by high temperature, plants have developed various defense mechanisms by regulating a variety of physiochemical mechanisms such as growth inhibition, leaf senescence, and basic physiological processes (Fahad et al., 2018), including a complex metabolic regulatory process called the heat shock response (Mittler et al., 2012).

Plants subjected to fairly common stresses, such as warmer summer conditions, respond by small but significant changes (Strasser and Tsimilli-Michael, 2001). Tests used to study changes induced by high temperature need to be very sensitive and informative. A very effective method is the measurement of Chla fluorescence, which can provide qualitative and quantitative information about the physiological state of photosynthetic organs, especially photosystem PSII. Numerous studies have shown that transient fluorescence kinetics is highly sensitive to various environmental changes (Kalaji et al., 2014a). Chlorophyll fluorescence kinetics is widely used in environmental science, agronomy, and plant science because of its fast, sensitive, and noninvasive properties (Strasser et al., 2004). Typical fast chlorophyll fluorescence induction kinetic curves have phases O, J, I, and P (Strasser and Govindjee, 1991), and in addition to these basic steps, some special stresses have been found under the L- (~150 μs) and K- (~300 μs) steps, as well as G- and H-steps between phases I-P (Strasser et al., 2004). The quantitative analysis of the fluorescence uptake kinetics O-J-I-P curve, called the JIP test based on the “biofilm energy flux theory” (Strasser and Strasser, 1995), translates the qualitative changes of the fluorescence uptake kinetics O-J-I-P curve into quantitative changes of selected phenomenological and biophysical-structural-functional parameters. proven to be a powerful and popular tool for quantifying PSII architecture and behavior (Tsimilli-Michael, 2019).

Peony (*Paeonia suffruticosa* Andr.) is one of the traditional flowers of China. It has a broad market prospect because of its tall, colorful and beautiful flowers. It can be used as potted flowers, cut flowers and horticultural materials to form a unique seasonal landscape (Li et al., 2011; Xue et al., 2018). Peony is suitable for cool climate and does not tolerate high temperature. In contrast, the summer in Jiangnan is hot and rainy, so peony growth and cultivation in Jiangnan is more influenced by climate (Zhang et al., 2015; Zhu et al., 2021). Summer is a critical period for rapid growth of peony leaves, nutrient accumulation and flower bud differentiation, but the high temperature in summer is the main reason that prevents peony from being widely cultivated in Jiangnan (Wang et al., 2012). Therefore, it is of great significance to study and master the growth and development rules of peony under high temperature stress to solve the problems of breeding of resistant peony cultivars, adjustment of variety structure and exploration of cultivation technology. However, recent studies have mainly focused on peony flower color (Gu et al., 2019; Guo et al., 2019) and seed oil extraction (Sun et al., 2017; Li et al., 2021). Effective protocols for micropropagation and the effects of different medium compositions and exogenous hormones on healing browning have also been investigated (Wen et al., 2016; Zhou et al., 2016). Few studies on heat tolerance of peony have been reported, mainly focusing on the photosynthetic characteristics and physiological and biochemical changes molecular mechanisms; the effect of exogenous hormone addition on high temperature tolerance of peony. It was found that high temperature stress inhibits the activity of superoxide dismutase, intensifies the production of H_2_O_2_ and malondialdehyde, inhibits the activity of PSI and PSII and causes damage to the photosynthetic machinery (Luo et al., 2011; Liu et al., 2012; Liu, 2019); exogenous abscisic acid and epilactone can improve the high temperature tolerance of peony (Wu et al., 2018; Ren et al., 2018); and heat shock proteins and heat tolerance genes were identified and verified (Zhang et al., 2015; Liu et al., 2019). Studies on the mechanisms of photosynthesis inhibition by high temperature and on the differences in tolerance to high temperatures in different peony cultivars have been limited so far. The aim of this study was to investigate the daily responses of two peony cultivars at the photosynthetic level under high temperature conditions. We evaluated and analyzed the photochemical adaptations of the investigated peony cultivars using photosynthetic parameters and JIP tests. To explore the causes of heat tolerance differences among different peony varieties, it provides the selection of heat resistant peony for suitable cultivation in Jiangnan region and the theoretical basis for the breeding of heat resistant varieties.

## Materials and methods

### Plant materials and treatments

In November 2020, four-year-old peony plants (‘Lu He Hong’ and ‘Zhi Hong’) with consistent growth and healthy were selected and planted in plastic pots with an upper diameter of 28 cm, a lower diameter of 19 cm and a height of 23 cm. In June 2021, the experimental material was domesticated using an artificial climate chamber for 7 days, with a 25°C/20°C day and night cycle, air humidity set at 70%, light intensity set to 4000lx, and a daily light time to dark time ratio of 14h/10h. On day 8, the peony plants were divided into two groups: ‘Lu He Hong’ and ‘Zhi Hong’, and three plants in each group were treated at 40°C/35°C daily cycle at high temperature, with environmental conditions consistent with domestication. In addition, samples were sampled at 0,2,4, and 6 days after treatment. During sampling, photosynthetic and chlorophyll fluorescence parameters were measured first measured, then leaves were taken for stress physiological indicators and protective enzyme activity assays. In addition, all the leaves were fully expanded, and the upper growth and healthy functional leaves (the first pair of leaves under the top bud) were selected.

### Determination items and methods

#### Stress physiological index and antioxidant enzyme activity measurement

The 0.1g leaves were first ground into fine powder in liquid nitrogen for subsequent indicator determination. The activities of malondialdehyde (MDA) content, superoxide dismutase (SOD, EC 1.15.1.1), and peroxidase (POD, EC1.11.1.7) were evaluated by the kit instructions (Suzhou Keming).

#### Determination of photosynthetic parameters

Photosynthetic parameters of peony leaves under high temperature treatment were measured in June 2021. The healthy functional leaves with similar growth in the middle and upper periphery of the plant (the first pair of leaves in the terminal bud) were selected, and LI-6400 portable photosynthesis instrument (LI-Cor6400XT PSC-4817, The net photosynthetic rate (Pn), stomatal conductance (Gs), intercellular CO_2_ concentration (Ci) and transpiration rate (Tr) of peony leaves were measured every morning (9:00-11:00 on sunny days), with three leaves per plant were measured in different cultivars in triplicate. The first determination of the blades should be numbered and marked for the next determination.

Stomatal limitation value (Ls)=1-Ci/Ca, where Ci denotes the intercellular CO_2_ concentration and Ca denotes the atmospheric CO_2_ concentration (CO_2a_). Water use efficiency (WUE) reflects the amount of CO_2_ assimilated per unit of water content transpired by the plant and is expressed as the ratio of net photosynthetic rate to transpiration rate: WUE=Pn/Tr.

#### Chlorophyll fluorescence measurements

The rapid chlorophyll fluorescence induction kinetic curve (OJIP curve) and related parameters of peony leaves under high temperature treatment were measured using a Handy PEA plant efficiency analyzer (Hansatech, UK) from 9:00 to 11:00 am. The dark responses were measured by clamping the same leaves with dark-adapted clamps for 30 min before the measurement. Three leaves for different varieties were repeated three times. After the first test, the leaves should be labeled for continuous determination.

#### Statistical analysis

Principal component analysis (PCA) is an effective tool for analyzing parameters and sample clustering, and is used to study the distribution patterns of OJIP parameters and treatments. In SPSS 25 software, the default setting is PCA/exploratory factor analysis using the maximum variance method. The process is as follows: Analysis → Dimensionality Reduction → Factor Run this process for factors. The rotated component matrices of the selected “JIP-test parameters” and the “principal component factor score table” were preformed on the two-dimensional plane consisting of PC1 and PC2. The posterior parameter distributions were plotted using the component matrix table.

SPSS 25 software was used for one-way ANOVA, Excel and Origin2021 software were used for data processing and mapping. Fisher’s least significant difference was used for mean comparison (P<0.05).

## Results

### Effects of high temperature stress on plant morphology of different peony cultivars

**Figure 1:**
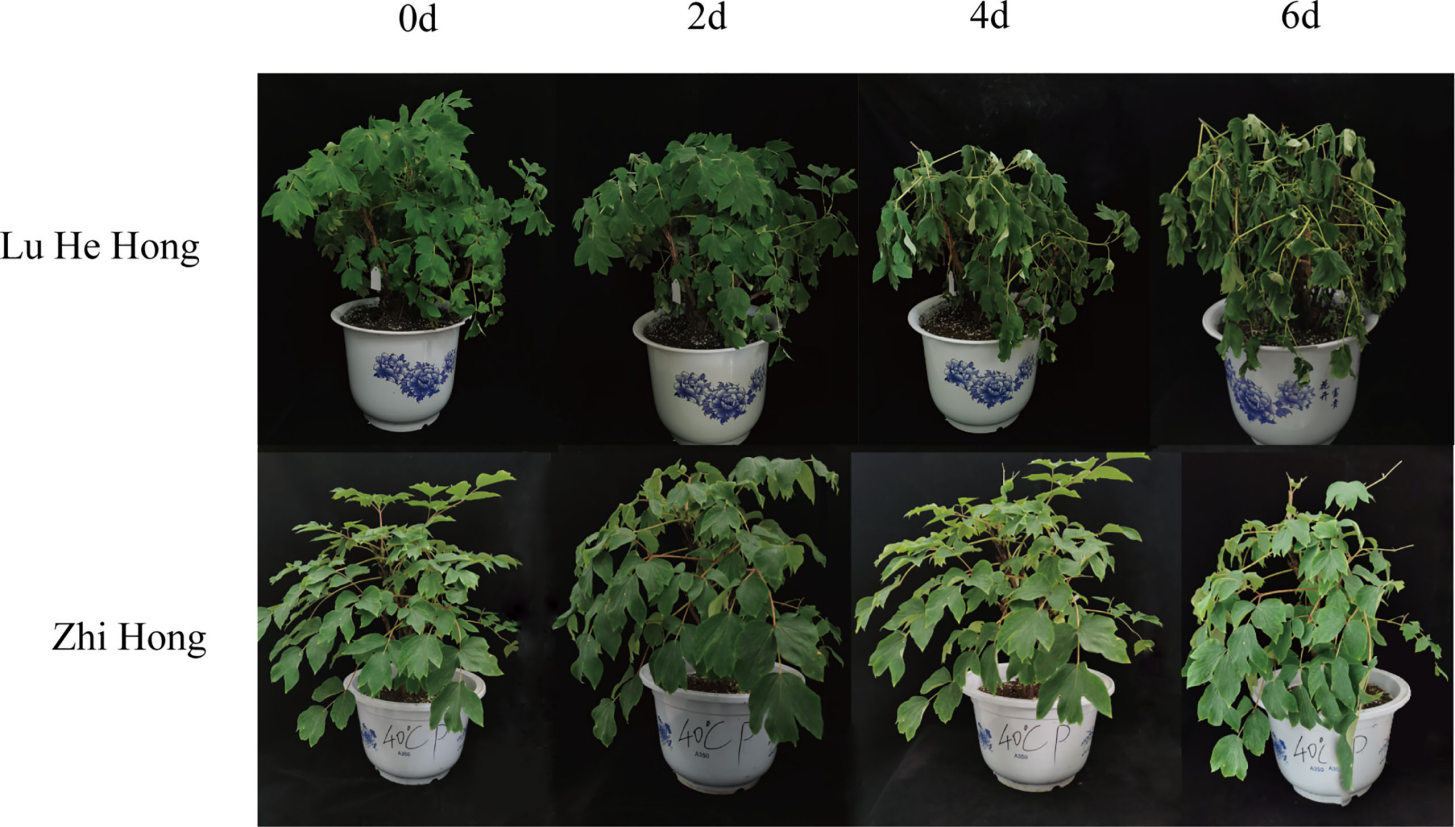
Effects of high temperature stress on morphology *P. suffruticosa* plantsof ‘Lu He Hong’ and ‘Zhi Hong’ cultivars.

In this study, morphological changes of different peony cultivars were observed under high temperature treatment at 40°C (**Figure 1**). The results showed that the growth status of peony changed significantly under high temperature treatment. Among them, ‘Lu He Hong’ changed greatly, the leaves began to wilt after 2 days, and the leaves shrank and branches drooped on the 4th day. By the 6th day, the leaves are yellow and dry, the branches droop severely, and the plant was wilted seriously. The ‘Zhi Hong’ variety showed strong heat resistance, with the extension of high temperature treatment time, the leaves kept vitality, only showed a slight droop of branches and yellow leaf tip morphological changes, until 25 days of high temperature treatment, the plants still maintained good vitality.

#### Effects of high temperature stress on oxidative stress of different peony cultivars

**Figure 2:**
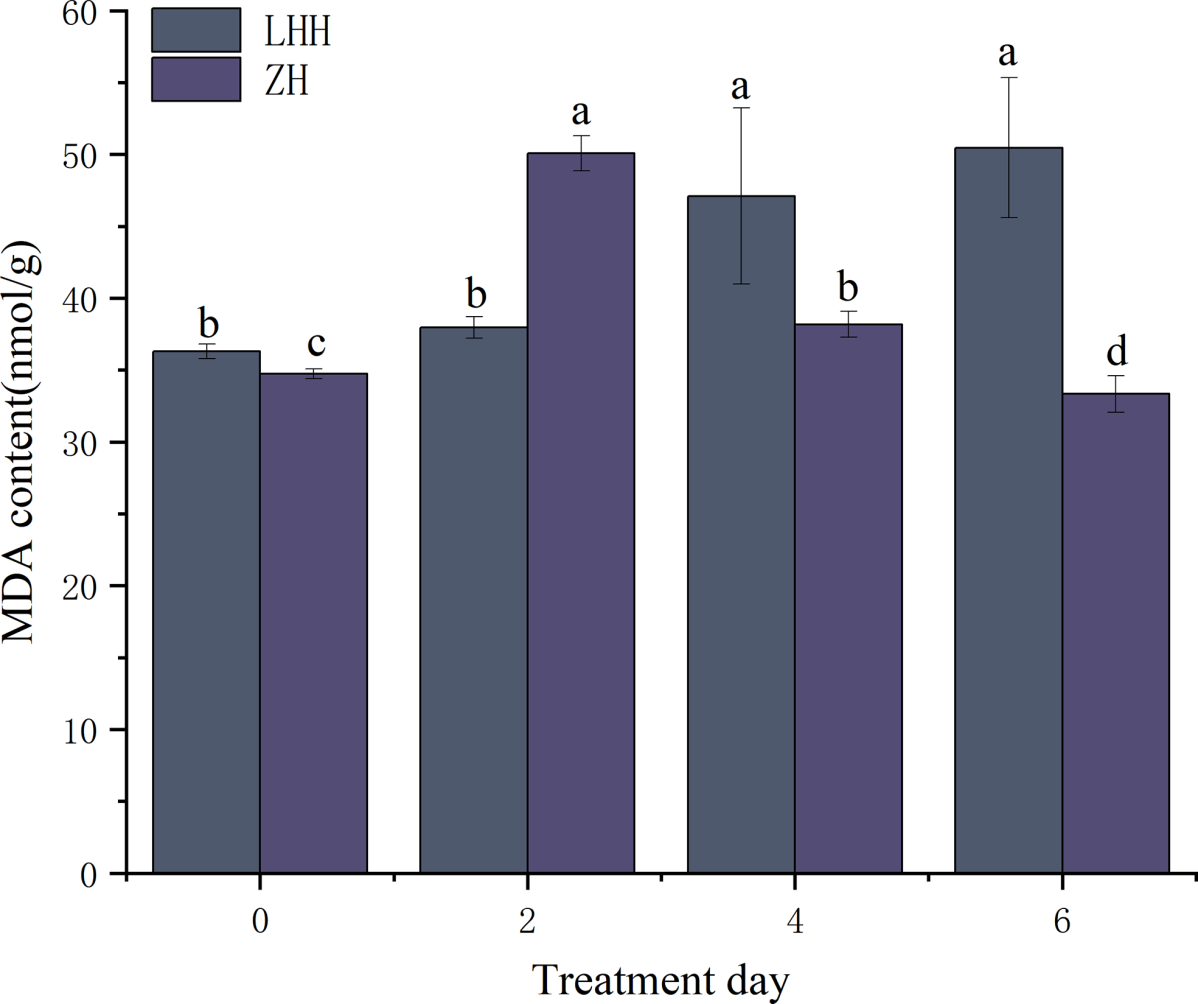
Effects of high temperature stress on oxidative stress in *P. suffruticosa* plants of ‘Lu He Hong’ (LHH) and ‘Zhi Hong’ (ZH) cultivars. Different letters in the same column indicate signifificant differences at the 0.05 level.

High temperature stress can cause membrane lipid peroxidation damage in peony (**Figure 2**). At 40°C, the content of MDA in Lu He Hong increased significantly and reached the peak on the 6th day, which was 39.05% higher than that in the untreated condition. However, MDA content in Zhi Hong leaves increased first and then decreased, reached the maximum on the second day, and then returned to the untreated level.

### Effects of high temperature stress on antioxidant enzymes of different peony cultivars

**Figure 3:**
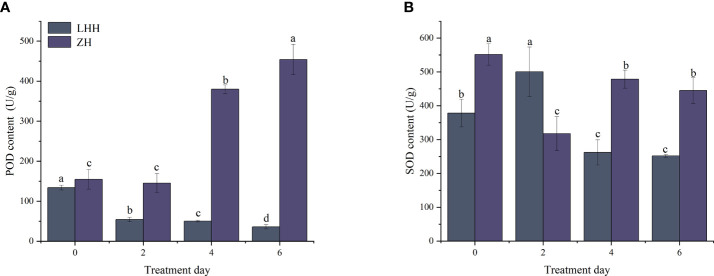
Effects of high temperature stress on oxidative stress in *P. suffruticosa* plants of 'Lu He Hong' (LHH) and 'Zhi Hong' (ZH) cultivars. **(A)** peroxidase (POD); **(B)** Superoxide dismutase(SOD). Different letters in the same column indicate significant differences at the 0.05 level.

High temperature stress had a significant effect on the activities of POD and SOD. As shown in **Figure 3A**, the POD activity of Lu He Hong showed a downward trend, decreasing by 59.50, 62.49 and 73.03% on day 2, day 4 and day 6. The POD activity of leaves after high temperature stress was always higher than that of leaves without high temperature stress, which was 1.94 times higher than that of leaves without high temperature stress. The SOD activity of Lu He Hong leaves increased first and then decreased with the extension of high temperature stress time, while Zhi Hong showed a trend of decreasing first and then increasing (**Figure 3B**).

#### Effects of high temperature stress on photosynthetic parameters of different peony cultivars

**Figure 4:**
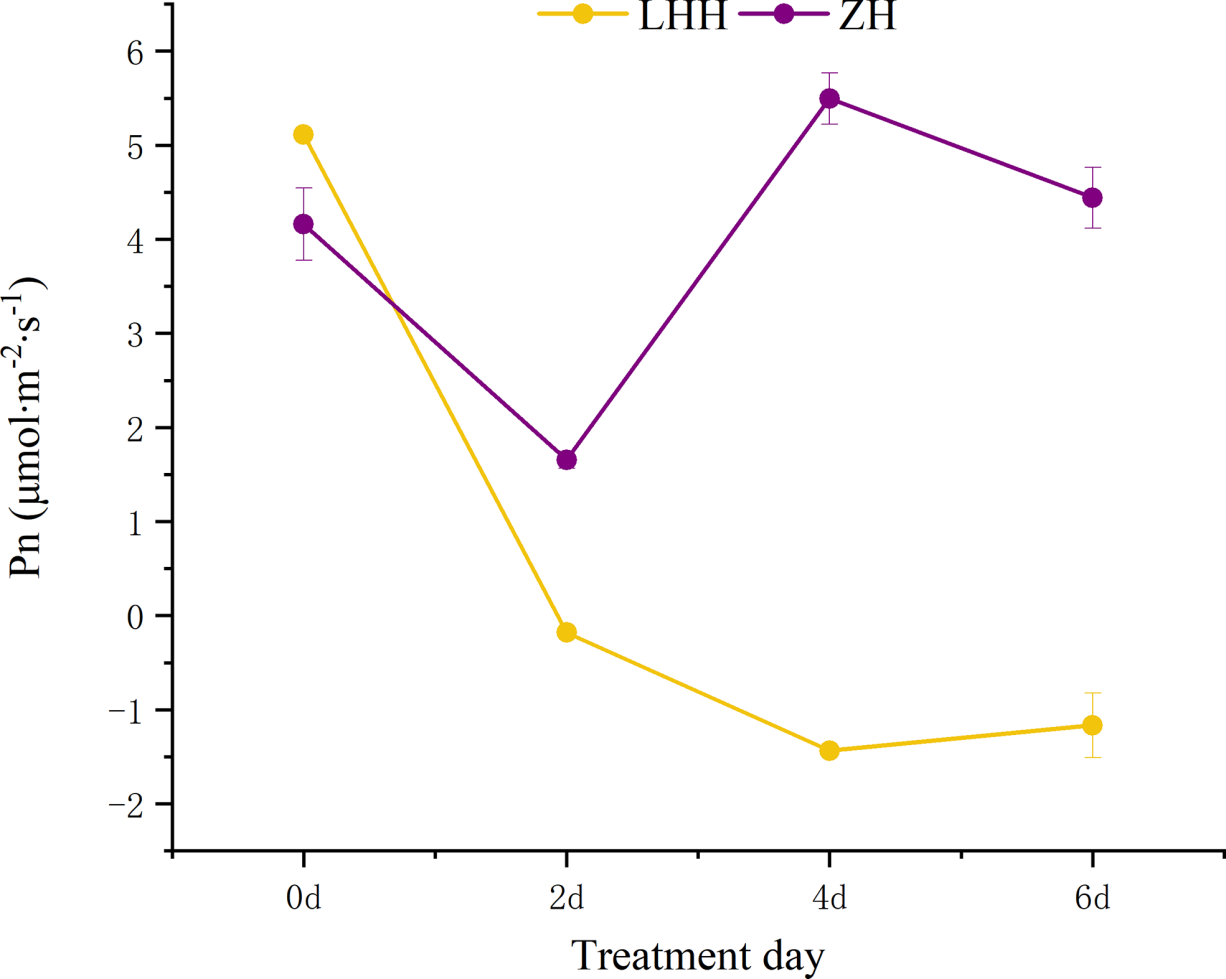
Effects of high temperature stress on Pn in *P. suffruticosa* plants of ‘Lu He Hong’ (LHH) and ‘Zhi Hong’ (ZH) cultivars. Different letters in the same column indicate signifificant differences at the 0.05 level.

**Figure 5:**
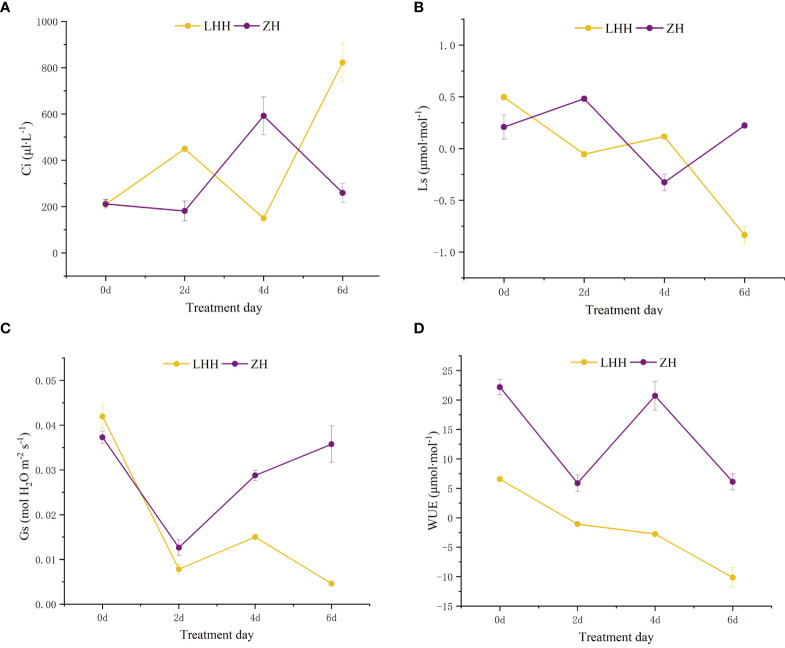
Effects of high temperature stress on gas exchange parameters in leaves in *P. suffruticosa* plants of ‘Lu He Hong’ (LHH) and ‘Zhi Hong’ (ZH) cultivars. Substomatal CO_2_ concentration (Ci; **A**); stomatal limitation value (Ls; **B**) stomatal conductance (Gs; **C**) and water use efficiency(WUE; **D**) in leaves of *P. lactifora* under high temperature treatment (HT).

High temperature effect the photosynthetic characteristics of leaves of different cultivars of peony (**Figures 4**, **5**). Under high temperature, the net photosynthetic rate of the two cultivars of peony decreased to different degrees, among which, the net photosynthetic rate of ‘Lu He Hong’ decreased seriously, with a large decrease in 0-2 days, and slowly decreased two days later. The ‘Zhi Hong’ began to rise after two days and returned to normal levels of net photosynthetic rate.

Under high temperature treatment, the intercellular CO_2_ concentration (Ci) and stomatal limit (Ls) of leaves of different peony cultivars changed significantly (**Figures 5A, B**). ‘Lu He Hong’ showed an upward trend first, then a downward trend and finally an upward trend; ‘Zhi Hong’ showed a trend of first decline, then rise and finally decline. The stomatal limit value was opposite to Ci.

The ‘Zhi Hong’ first decreased and then increased, and the stomatal conductance began to increase after the second day. ‘Lu He Hong’ showed a trend of first decline, then rise and finally decline (**Figure 5C**).

Water use efficiency (WUE) is a key physiological indicator to judge whether plant growth is under stress. In this study, it was found that high temperature stress had an impact on water use efficiency of different peony cultivars (**Figure 5D**). The water use efficiency of leaves of ‘Lu He Hong’ decreased gradually with the extension of high temperature treatment time. Although the water use efficiency of ‘Zhi Hong’ fluctuated, it was always higher than that of ‘Lu He Hong’.

### Effects of high temperature stress on chlorophyll fluorescence of different peony cultivars

**Figure 6:**
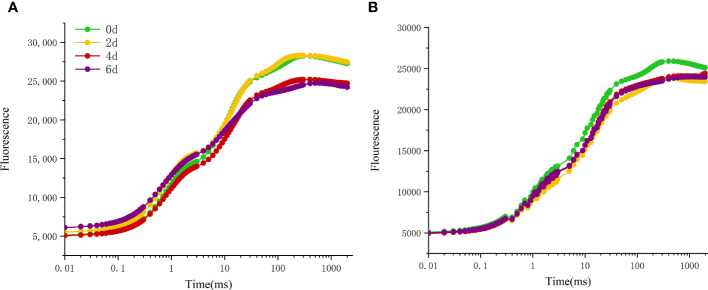
Effects of high temperature stress on OJIP curve in *P. suffruticosa* plants of ‘Lu He Hong’ (LHH) and ‘Zhi Hong’ (ZH) cultivars. **(A)** ‘Lu He Hong’; **(B)** ‘Zhi Hong’.

By constructing variable fluorescence curves, the changes of photosynthetic properties of different peony cultivars during leaf natural senescence were studied (**Figure 6**). The results showed that with the extension of high temperature treatment time, the fluorescence values of all cultivars of peony decreased in different degrees. The fluorescence value of ‘Lu He Hong’ decreased greatly (**Figure 6A**), while that of ‘Zhi Hong’ did not decrease significantly (**Figure 6B**).

**Figure 7:**
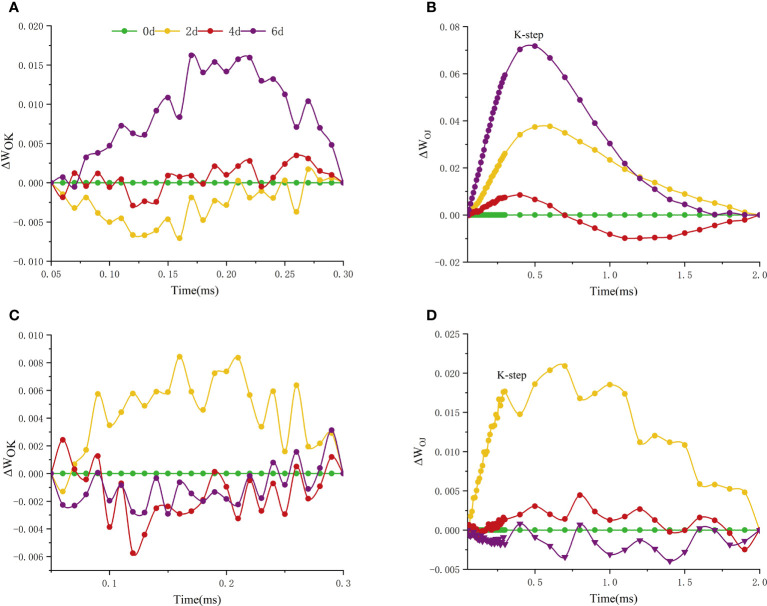
Effects of high temperature stress on the O-K and O-J phase kinetic curves in *P. suffruticosa* plants of ‘Lu He Hong’ (LHH) and ‘Zhi Hong’ (ZH) cultivars. **(A, C)** The O-K phase dynamics curve of ‘Lu He Hong’ and ‘Zhi Hong’ (double normalized by O-step (50 µs) and K-step (300 µs) to show L-band); **(B, D)** the O-J phase dynamics curve of ‘Lu He Hong’ and ‘Zhi Hong’ (double normalized by O-step (50 µs) and J-step (2 ms) to show K-band).

In measurements, an additional step labeled K and L steps appeared at 300ms and 150ms of the fluorescence transient, respectively. The appearance of L and K bands at high temperature reflects the transient values of these grades, with significant differences among cultivars in L and K bands (**Figure 7**). L-peak can indicate aggregation between different components of PSII or energy transfer connectivity between antenna pigment and RCs, the active reaction center of PSII. The L-band of ‘Lu He Hong’ presented a negative transient value at 0-4 days of high temperature treatment, and a positive L-band at 6 days of high temperature treatment (**Figure 7A**). The L-band of ‘Zhi Hong’ showed a positive transient value when it was exposed to high temperature for 2 days, and a negative transient value when it was exposed to high temperature for 4-6 days (**Figure 7C**). The presence of K-band indicates the inactivation of OEC on the donor side of PSII. K-band is often observed in plants exposed to high temperatures and is an indicator of OEC damage. The K-band of ‘Lu He Hong’ showed a positive and rising trend (**Figure 7B**). After 2 days of high temperature treatment, the K-band of ‘Zhi Hong’ showed a positive value, and 4-6 days, the K-band showed a negative transient value (**Figure 7D**).

**Figure 8:**
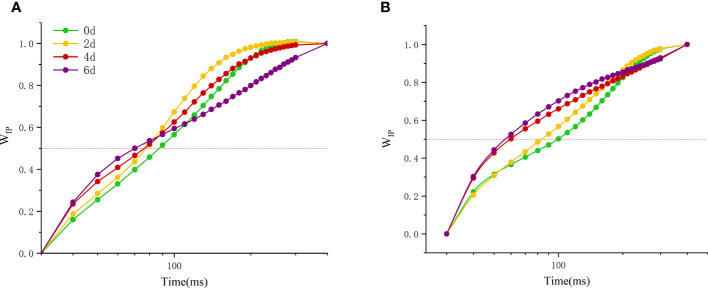
Effect of high temperature stress on the kinetic curve of I-P phase in *P. suffruticosa* plants of ‘Lu He Hong’ (LHH) and ‘Zhi Hong’ (ZH) cultivars. **(A)** ‘Lu He Hong’; **(B)** ‘Zhi Hong’.W_IP_== (F_t_ − F_I_)/(F_P_− F_I_), I indicates the I-step at about 30 ms; P indicates the P-step at about 300 ms.

Another fluorescence normalized W_IP_ (normalized by I- and P-step) ascent kinetics, W_IP_=0.5 can be used to reflect the reduction rate of the electron receptor at the end of the PSI receptor side (**Figure 8**). With the extension of high temperature time, the W_IP_ of the two peony cultivars changed significantly, and there were significant differences among the cultivars. The time of W_IP_ value of 0.5 was gradually decreased. The initial value of ‘Lu He Hong’ (**Figure 8A**) is smaller than that of ‘Zhi Hong’ (**Figure 8B**), but the variation range of ‘Lu He Hong’ is smaller than that of ‘Zhi Hong’.

**Figure 9:**
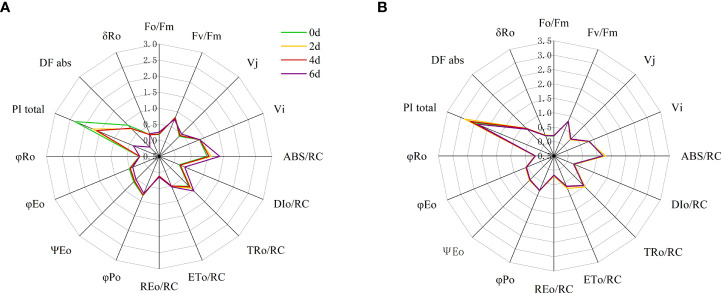
Radar plots of leaf structural and functional JIP test parameters of peony cultivars **(A)** ‘Lu In this manusript, the comments are made. He Hong’, **(B)** ‘Zhi Hong’at 0, 2,4 and 6 days after heat treatment. Quantum ratio used for heat dissipation (φ_Do_=1-φ_Po_=(F_o_/F_m_)); the maximum quantum yield of the initial photochemical reaction (φ_Po_=≡ TR_0_/ABS = [1 – (F_o_/F_m_)] = F_v_/F_m_)); quantum yields of electron receptors at the end of the reduced PSI receptor side (φ_Ro_== RE_0_/ABS = φ_Po_ (1 – V_I_)); quantum yield for electron transport (ET)(at t=0) (φ_Eo_≡ET_0_/ABS= [1−(F_o_/F_m_)]ψ_Eo_); the efficiency of electron transfer other than Q_A_ driven by a single exciton captured by the active reaction center (at t=0) (Ψ_Eo_= ET_0_/TR_0_ = [1 – (F_J_/F_m_)] = 1- V_J_); Ratio of electrons from the reductive PSI receptor side terminal electron acceptor to electrons from the electron transport chain (δ_R0_=RE_0_/ET_0_=(1-V_I_)/(1-V_J_)); absorption-based driving force expressed from the absorption of photons to the reduction of the electron transfer chain (DF_abs_); Performance index (potential) for energy conservation from exciton to the reduction of PSI end acceptors (PI_total_≡PI_abs_[δ_Ro_/(1−δ_Ro_)]); absorption flux (of antenna Chls) per RC (ABS/RC=M_O_ (1/V_J_)(1/φ_Po_)); trapped energy flux per RC(at t=0) (TR_0_/RC=M_O_ (1/V_J_)); electron transport flux (further than $Q_{A}^{-}$ ) per RC(at t=0) (ET_0_/RC=M_O_ (1/V_J_)ψ_Eo_); dissipated energy flux per RC(at t=0) (DI_0_/RC=(ABS/RC)-(TR_0_/RC)); specific electron flux per unit PSII active reaction center for reduction of PSI terminal electron acceptor (at t=0) (RE_0_/RC=(1-V_I_)(M_O_/V_J_)).

Significant differences were observed between most JIP test parameters (fluorescence index (F_o_/F_m_, F_v_/F_m_), relative variable fluorescence (V_J_, V_I_), quantum yield and efficiency (φ_P0_, φ_R0_, φ_E0_, ψ_E0_, and δ_R0_), specific energy fluxes, and phenomenological fluxes (ABS/RC, TR_0_/RC, ET_0_/RC, DI_0_/RC and RE_0_/RC) and performance metrics (DF_abs_ and PI_total_) were significantly different (**Figure 9**; **Table S1**). At two days of high temperature treatment, V_J_, ABS/RC, TR_0_/RC of ‘Lu He Hong’ leaves increased significantly; ψE0, DF_abs_ and PI_total_ decreased significantly; Fo/Fm, Fv/Fm, V_I_, DI_0_/RC, ET_0_/RC, RE_0_/RC, φ_P0_, φ_R0_, φ_E0_, δ_R0_ did not change significantly; on day 4, compared with day 2, DF_abs_ and PI_total_ increased significantly, while the remaining parameters did not change significantly; at 6^th^ day, compared with day 4, V_J_, ABS/RC, TR_0_/RC, Fo/Fm, DI_0_/RC, RE_0_/RC, δ_R0_ showed a significant increase; ψ_E0_, φ_P0_, φ_E0_, Fv/Fm, DF_abs_ and PI_total_ decreased significantly; V_I_, ET_0_/RC, and φ_R0_ did not change significantly (**Figure 9A**).

‘Zhi Hong’ showed no significant changes in φ_E0_, φ_R0_, δ_R0_, DF_abs_ and PI_total_, significant increases in Fo/Fm, ABS/RC, DI_0_/RC, TR_0_/RC, ET_0_/RC, RE_0_/RC, ψ_E0_, and significant decreases in Fv/Fm, φ_P0_, Vj, Vi decreased significantly; at day 4, compared with day 2, Fo/Fm, Fv/Fm, φ_P0_, V_J_, V_I_, ψ_E0_, φ_E0_, φ_R0_, δ_R0_, DF_abs_, and PI_total_ did not change significantly; ABS/RC, DI_0_/RC, TR_0_/RC, ET_0_/RC, RE_0_/RC occurred a significant decrease and returned to the non-high temperature treatment to the same level without high temperature treatment (**Figure 9B**).

**Figure 10:**
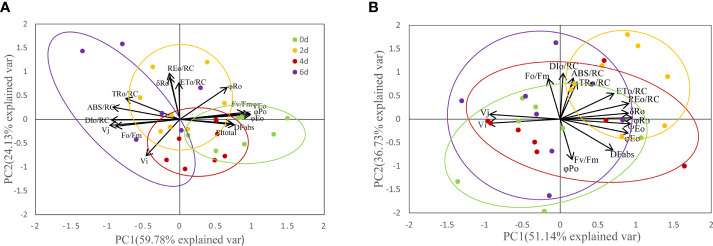
PCA of JIP parameters of Peony leaves treated at 40 °C for different time. **(A)** ‘Lu He Hong’; **(B)** ‘Zhi Hong’.

To further evaluate the results, we conducted PCA analysis based on the collected JIP test parameters measured for each leaf during the high temperature treatment (**Figure 10**). The cumulative contribution values of PC1 and PC2 for all parameters analyzed in ‘Lu He Hong’ were 59.78% and 24.13%, with a joint cumulative contribution value of 83.91%; the cumulative contributions of PC1 and PC2 in ‘Zhi Hong’, the cumulative contribution values of PC1 and PC2 were 51.14% and 36.73%, respectively, and the joint cumulative contribution value was 87.86%. This indicates that the two-component factors obtained from the principal component analysis have a good separation description of the selected parameters. Through the principal component analysis, we found that the selected JIP test parameters formed three well-separated clusters. Among them, for ‘Lu He Hong’, the formation of PC1 was mainly due to the difference of PSII status parameters; the formation of PC2 was due to PSI parameters φ_R0_, δ_R0_, and PI_total_ (**Figure 10A**). While PC1 of ‘Zhi Hong’ corresponds to PSI activity, the higher value indicates higher PSI performance; PC2 corresponds to PSII activity, the higher PSII value, the higher PSII performance, representative parameters include Fv/Fm, DF_abs_, φ_Eo_ and ABS/RC (**Figure 10B**). Cluster analysis of the experimental results showed that with the deepening of stress, the location of clustering also changed. PCA of all peony cultivars showed that the ellipsoid separation degree of ‘Lu He Hong’ was higher, indicating the severity of stress damage. The ellipsoid of ‘Zhi Hong’ has a good overlap area, indicating that the stress response is not obvious. Among them, the stress degree of ‘Lu He Hong’ was the most serious, showing high PSII performance at day 0. The PSII performance was damaged and PSI performance was improved after two days high temperature stress, while PSI performance was decreased after four days of high temperature treatment. On the sixth day, PSII performance was seriously damaged and PSI performance was improved. The stress response of ‘Zhi Hong’ was mild, and the properties of PSI and PSII did not change significantly.

## Discussion

Reduction in plant photosynthesis and inhibition of photosynthesis are early indicators that plants are in a stressful environment (Olsovska and Brestic, 2001). In this study, we followed morphological changes as well as changes in photosynthetic parameters and OJIP chlorophyll fluorescence kinetics in two different peony cultivars under high temperature stress, and the study showed that high temperatures produced different degrees of damage to different cultivars of peony. In addition, the physiological and biochemical characteristics of peony were significantly affected by high temperature stress. Previous studies have shown that weather disasters will have a deeper impact on the photochemical performance of plants as future climate changes are more intense and longer duration of high temperatures (Kharshiing and Sinha, 2016).

### Effect of high temperature stress on the physiological and biochemical indexes of different peony cultivars

As a form of high temperature stress, oxidative stress also has adverse effects on plant growth and development. In addition, excessive ROS accumulation caused peroxidation damage of membrane lipid in peony, and MDA content increased significantly with the development of high temperature stress, which may be due to the decrease of antioxidant enzyme activity. MDA is the final decomposition product of membrane lipid peroxidation. Under high temperature stress, the accumulation of MDA content will damage the structure and function of plant cell membrane. Antioxidant systems play an important role in protecting plants from the negative effects of ROS (Srivalli et al., 2003). The main antioxidant enzymes to eliminate ROS contain SOD, POD and so on. In this study, POD and SOD activities in peony leaves increased under high temperature stress, mainly because the plants initiated stress response and self-protection mechanism, adapted to the external environment by regulating antioxidant enzyme activities, and re-established the balance between production and removal of reactive oxygen species (Wang & Li, 2006). Regulation of ROS is the signal of the reactive oxygen species gene network in plant cells, which then regulates the activity of antioxidant enzymes. However, in the process of long-term high temperature stress, when the adaptation and self-protection capacity of plants is exceeded, the antioxidant enzyme activities are inhibited (Scalet et al, 1995). The POD activity of ‘Lu He Hong’ showed a decreasing trend, while the SOD activity increased first and then decreased. However, the activity of ‘Zhi Hong’ POD increased continuously, while SOD decreased first and then increased. It can be seen that the antioxidant system of ‘Lu He Hong’ is unstable, and the heat resistance of ‘Zhi Hong’ is strong.

### Effect of high temperature stress on photosynthetic characteristics of different peony cultivars

Photosynthesis is the main factor affecting plant growth and development, and is the element most susceptible to high temperature stress (Lu et al., 2017; Du et al., 2022). When the ambient temperature is higher than the optimal temperature of the plant, the chloroplast structure will be damaged, and the photosynthetic performance will be reduced, and the relevant photosynthetic parameters will be changed (Chen et al., 2012). Net photosynthetic rate can directly represent the photosynthetic capacity of a single leaf (Jiang et al., 2004). Zhang et al. (2022) showed that under high temperature stress, the net photosynthetic rate of plants decreased continuously, but there were significant differences among different cultivars. This study showed that the net photosynthetic rate of ‘Lu He Hong’ decreased with the deepening of stress degree, and that of ‘Zhi Hong’ decreased first and then increased to the normal level within six days under temperature stress, indicating that high temperature stress has a negative effect on the photosynthesis of peony plants, which is consistent with (Zhang et al., 2022). The research is consistent that ‘Zhi Hong’ has good heat resistance and can quickly adapt to heat stress. Stomatal restriction and non-stomatal restriction are one of the important factors leading to the decline of photosynthetic capacity of plants. Stomatal restriction and non-stomatal restriction factors causing the decrease of photosynthetic rate can be judged according to the change direction of Ci and Ls in leaves (Xu, 2013). In this study, the Pn decline of ‘Lu He Hong’ was firstly dominated by non-stomatal limiting factors, then by stomatal factors and finally by non-stomatal limiting factors, while the Pn decline of ‘Zhi Hong’ was firstly dominated by stomatal limiting factors, then by non-stomatal limiting factors and finally restored to stomatal limiting factors. When stomatal limitation factors are dominant, stomatal conductance of leaves decreases and CO_2_ entering stomata decreases, which cannot meet the requirements of photosynthesis. However, the decrease of Pn not dominated by stomatal limitation is due to the increase of leaf temperature, the decrease of chloroplast activity and Rubisoo activity, and the decrease of RuBP carboxylase regeneration capacity, leading to the decrease of photosynthetic capacity of leaves (Gao et al., 2018). Therefore, the reason for the decrease in the net photosynthetic rate of 'Lu He Hong' in this study may be mainly due to the irreversible damage of the chloroplast structure, whereas the high tempeature resistant 'Zhi Hong' has a more stable chloroplast structure that can adapt to short-term high temperature stress. Stomata is a channel for gas exchange between plants and the environment. Stomatal conductance (Gs) represents the degree of stomatal opening and is a major factor in photosynthesis, respiration and transpiration rate of plants (Miner et al., 2017). In this study, Gs of ‘Lu He Hong’ decreased with the deepening of high temperature stress, indicating that high temperature stress had a negative impact on its stomatal conductance, which was consistent with the research results of Sarwar et al. (2019). ‘Zhi Hong’ results in stomatal closure during short-term heat treatment to prevent leaf dehydration, and with the deepening of high temperature stress, stomatal conductance increases and leaf temperature decreases to balance the ambient temperature. WUE reflects the amount of CO_2_ assimilated by plants per unit water content of transpiration and is a comprehensive indicator to evaluate the adaptability of plants to the environment. The larger WUE value is, the less water consumed by fixed unit mass CO_2_ (Zhang and Shan, 2002). Hao et al. (2017) found that water utilization rate of Peony decreased significantly under high temperature stress. In this study, ‘Lu He Hong’ decreased significantly with the prolongation of high temperature stress, which was consistent with previous studies. However, the WUE of ‘Zhi Hong’ is always higher than that of ‘Lu He Hong’, indicating that ‘Zhi Hong’ has full water utilization, can consume less water content and produce more nutrients, which is consistent with the research results, and ‘Zhi Hong’ has the characteristics of thermal stability.

### Effect of high temperature stress on chlorophyll fluorescence of different peony cultivars

The rapid chlorophyll fluorescence induction kinetics curve refers to the fluorescence change process from O point to P point, which mainly reflects the initial photochemical reaction of PSII and the changes in the structure and state of photosynthetic apparatus (Krause and Weis, 1991). When plants are subjected to high temperature stress, K-band (300μs) will appear. This is because the donor side of PSII is damaged at high temperature. After a very short period of time, the chlorophyll fluorescence intensity increases rapidly, resulting in the transformation of OJIP curve into OKJIP curve (Chen et al., 2004), and the emergence of K-band indicates that OEC is damaged (Du et al., 2011). The positive K-band is caused by the decrease of the electron transfer rate at the donor side or the increase of the electron outflow rate at the acceptor side due to the inactivation of OEC. The balance between electron transfer and further electron transfer to Q_A_ is impaired, resulting in RC damage. The negative K-band is caused by an increase in the rate of electron transfer from the donor side or a decrease in the rate of electron outflow from the acceptor side (electron transfer delay) (Kalaji et al., 2014b). In this study, the parameter ΔW_OJ_ was introduced to analyze the changes of donor side and OEC of PSII of different peony cultivars, and there were significant differences among cultivars. The K-Band of ‘Lu He Hong’ increased with the extension of high temperature stress time, indicating that the stress caused damage to the oxygen release complex of the variety, and reduced the electron transfer rate of the PSII donor side. ‘Zhi Hong’ showed significant positive value of K-Band for two days under high temperature stress, indicating that the OEC was slightly damaged due to high temperature stress response during short-term high temperature treatment. K-band is negative after two days of high temperature treatment, and negative K-band has been widely used in recent literature as a marker of tolerance to various stresses(Mathur et al., 2011), which is consistent with ‘Zhi Hong’ showing good heat resistance. L-band can reflect the aggregation between different components of PSII or the energy transfer connectivity between antenna pigment and RCs, the active reaction center of PSII (Srivastava et al., 1997; Stirbet, 2013). A positive L-band indicates dissociation of the antenna pigment complex with greater distance between PSII antennas and therefore less efficient energy exchange, while a negative L-band indicates greater grouping and more efficient energy exchange between neighboring PSII units (Tsimilli-Michael, 2019; Kalaji et al., 2018). L-band was negative within 4 days of high temperature treatment, indicating that it adapted to the damage caused by high temperature by increasing energy exchange under high temperature stress. Meanwhile, L-band showed a positive value by day 6, indicating that at this time, the energy exchange of 'Lu He Hong' decreased sharply, which may be irreversible damage caused by long periods of high temperature stress. In contrast, the L-band of ‘Zhi Hong’ only showed positive values at two days under high temperature, after which the L-band presented negative values, which indicated that ‘Zhi Hong’ had a better ability to adapt to heat stress and resist the damage caused by high temperature by increasing energy exchange. W_IP_ = 0.5 (half time of the rising curve) can be used to reflect the reduction rate of the electron acceptor pool at the end of the PSI (Guo et al., 2020) and also the half-life of the rising curve (Xu et al., 2021). It can be seen that both peony cultivars in this study were subjected to a significant increase in the reduction rate of the PSI terminal electron acceptor pool and a significant reduction in the half-life time under high temperature treatment.

### Effect of high temperature stress on chlorophyll fluorescence parameters of different peony cultivars

Analysis using the JIP test parameters allowed us to assess the effect of different stresses on the efficiency and flux of electrons as well as the efficiency and flux of energy around PSI and PSII (Maxwell and Johanson, 2000). To quantify the changes in light absorption, utilization and chemical energy conversion and to analyze the fluorescence transients, the JIP test was performed in this study and the parameters shown on the radar plot provide a clearer picture of the changes under high temperature stress in different peony cultivars. The most significant change in JIP parameters was in the ‘Lu He Hong’, which is consistent with the change in its OJIP phase and the early appearance of L-band and K-band changes. ‘Lu He Hong’ on the second day of high temperature treatment only showed significant changes in Vj, PI_total_, DF_abs_, ABS/RC, TR_0_/RC, and ψ_E0_ parameters changed significantly, although other indicators for significant changes, the appearance of L-band and K-band is an early indicator of impaired photochemical properties (Viljevac Vuletić and Španić, 2019). Fv/Fm (or φ_P0_) is an indicator of PSII light energy conversion rate (Cai et al., 2017), and the decrease of Fv/Fm is an important indicator of photoinhibition (Strasser et al., 2004). In this study, Fv/Fm of the two cultivars significantly decreased under high temperature stress, indicating that high temperature would produce photoinhibition on peony and affect photosynthesis. J phase relatively variable fluorescence (V_J_) represents the probability that a captured exciton moves an electron from the main acceptor (Q_A_) into the electron transport chain. When Q_A_ reoxidation is restricted, the V_J_ value increases, resulting in the accumulation of Q_A_ reduction and the reduction of electron transport. The I step (V_I_) decreases after high temperature and excessive light exposure, indicating that electron transport is lower than Q_A_ due to higher levels of Q_B_- non-reduction centers (Mihaljević et al., 2020). Under high temperature stress, the V_J_ of ‘Lu He Hong’ showed an increasing trend, while φE0 decreased, indicating that the PQ pool had poor electron acceptance ability under high temperature stress, resulting in limited electron transfer ability of Q_A_ from acceptor to Q_B_, thus the electron transfer from pheo to Q_A_ resulted in $Q_{A}^{-}$ , This eventually leads to a large accumulation of $Q_{A}^{-}$ (Kalaji et al., 2014b) and a decrease in the efficiency of PSII electron transfer (Öquist et al., 1992). V_J_ of ‘Zhi Hong’ decreased on the second day and electron transfer rate increased, and V_J_ returned to the original level after two days of stress, indicating that the variety has strong heat resistance and can better adapt to the environment under short-term high temperature stress. The relative fluorescence of I-phase did not change significantly in ‘Lu He Hong’, and ‘Zhi Hong’ decreased on the second day under high temperature stress but returned to normal level later. Blockage of electron transport leads to leakage of electrons in the electron transport chain, which attack intracellular O_2_ and generate ROS, especially singlet reactive oxygen species, which play an important role in membrane lipid peroxidation (Chalanika et al., 2017). Therefore, the leaves of peony under high temperature stress will inevitably lead to the accumulation of ROS and damage to cell membrane structure, which is consistent with the significant increase of MDA in our results. Among them, the increase of MDA of ‘Lu He Hong’ is more significant than that of ‘Zhi Hong’, which is positively correlated with the electron transport efficiency.

DF_abs_ and PI_total_ can be found to be the parameters with significant changes in the pre-tolerant ‘Lu He Hong’. The total performance index (PItotal) is the most prevalent and sensitive parameter in JIP testing, as it reflects changes in photosynthetic electron transport activity outside PSII, intersystem electron transport and PSI processes (Stefanov et al., 2011). A reduction in PI_total_ has been found in apple cultivars under high temperature stress (Mihaljević et al., 2020), indicating that high temperature stress negatively affects PSI in peony. In this experiment, with the deepening of high temperature stress, the DFabs and PItotal of ‘Lu He Hong’ decreased significantly, while ‘Zhi Hong’ did not change significantly, which means that the PSI of ‘Lu He Hong’ was unstable under high temperature stress. Performance indicators of the reduced overall energy flow from the excitons to the reduced PSI-terminal receptors coincide with the reduced electron acceptor state (R0) at the lateral end of the PSI receptors. The fast chlorophyll fluorescence induction kinetic curve than activity parameters, including light energy absorbed per reaction center (ABS/RC), energy captured per reaction center for Q_A_ reduction (TR_0_/RC), energy captured per reaction center for electron transfer (ET_0_/RC), and energy dissipated per reaction center (DI_0_/RC), can accurately reflect the plant photosynthetic organ absorption, conversion and dissipation of light energy by plant photosynthetic organs (Strasser et al., 2001; VanHeerden et al., 2004). In this study, the ABS/RC, TR_0_/RC and DI_0_/RC of ‘Lu He Hong’ and ‘Zhi Hong’ were significantly increased, but the specific activity parameters of ‘Zhi Hong’ returned to normal level after two days of high temperature treatment, which indicated that after the inactivation or cleavage of some reaction centers per unit area of the leaves triggered by high temperature stress, the efficiency of the remaining active reaction centers could be promoted to better dissipate the energy in the electron transport chain, which might be a self-protection mechanism of peony leaves.ABS/RC represents the effective antenna size of the active RC, which is influenced by the proportion of active and inactive reactive centers, and when the number of inactive centers increases, so does the proportion of ABS/RC (Mathur et al., 2011). This parameter value was higher in two peony cultivars, suggesting that PSII may have more efficiency of energy capture and a higher increase in inactive centers compared to ‘Zhi Hong’. The increase in ABS/RC may be due to a reduced ratio of active RCs and an increase in absorbed light energy, which may be related to the deactivation of RCs or an increase in antenna size. Previous studies (Tao et al., 2022) showed that in general, RCs inactivation of plants is related to photoinhibition, that is, when the amount of light absorbed by plants exceeds the amount of light that can be utilized by their own photosystem, the photosynthetic function of plants decreases. Derks et al. (2015) found that when photoinhibition occurs in plants, the plants actively shut down some of the RCs and release the excess absorbed light energy through heat. The increase in this parameter was greater in ‘Lu He Hong’ than in ‘Zhi Hong’, suggesting that high temperature is more damaging to ‘Lu He Hong’ RCs. The increase in ABS/RC values is accompanied by an increase in the parameter TR0/RC, which represents the maximum speed at which RC can trap excitons (Stirbet and Strasser, 1996). TR_0_/RC and DI_0_/RC in the ‘Lu He Hong’ had a significant rise in RC, Fv/Fm significantly reduced, that all $Q_{A}^{-}$ were reduced, can no longer oxidation. When $Q_{A}^{-}$ reoxidation was suppressed, electron transfer to $Q_{A}^{-}$ cannot effectively Q_B_, because the active center can’t capture a large number of photons, so the excess photon is known as the dissipation energy (Mathur et al., 2013). It was also observed that the efficiency of individual electrons from the interphotosystem electron transport chain to the terminal electron acceptor on the PSI acceptor side (δ_R0_) of the more heat-tolerant ‘Zhi Hong’ did not change under high temperature treatment, whereas there was a significant increase in the less heat-tolerant ‘Lu He Hong’. It may be due to the flow of electrons between the two photosystems, the flow of PSI terminal receptors are slightly accelerated and PSI activity is increased (Blankenship, 2014).

PCA was used to evaluate the PSI and PSII activities of peony leaves by multi-parameter analysis with more systematic and comprehensive effects. The results showed that several parameters related to PSI and PSII activities were influenced by temperature. In this study, we used a new method and idea to analyze the differences in photosynthetic and fluorescence-related parameters of leaves of two peony cultivars with different heat tolerance, and made a comprehensive evaluation of the photosynthetic performance of peony leaves to explore the reasons for the differences in heat tolerance among different peony cultivars. The results of this study provide a theoretical basis for the screening of heat-resistant peonies suitable for cultivation in Jiangnan area and for the selection and breeding of high temperature resistant varieties.

## Conclusion

Peony has a long history of cultivation in China and has high ornamental value. However, due to the high temperature and rainy summer in southern China and global warming, it is difficult for peony to move southward. Different peony cultivars in order to understand the heat photosynthetic performance and physiological and biochemical changes, photosynthesis of two cultivars of peony and chlorophyll fluorescence, physiological and biochemical indexes were analyzed, and discussed the causes of differences in heat resistance between different cultivars of peony, for screening suitable for cultivation in the south of the Jiangnan the heat resistance of peony, as to provide theoretical basis for the breeding of heat resistant cultivars. Studies have shown that the more heat-resistant ‘Zhi Hong’ has a more stable cell membrane, which is not easy to be oxidized, and the antioxidant system is also more stable. In addition, ‘Zhi Hong’ with strong heat tolerance has stable green cell activity, has higher water utilization rate and can produce more nutrients, and can maintain high photosynthetic performance under high temperature stress, while ‘Lu He Hong’ photosynthetic capacity decreases continuously under high temperature stress, and the photosynthetic mechanism is unstable and vulnerable to damage. JIP-test analysis showed that OEC, PSI and PSII reaction centers and RCs of ‘Zhi Hong’ during high temperature stress are slightly damaged to adapt to the high temperature stress environment by increasing greater grouping and more efficient energy exchange between adjacent PSII units and maintaining the balance between electron transport, and ‘Lu He Hong’ performs poorly in these aspects, consistent with its poor heat resistance.

## Data availability statement

The original contributions presented in the study are included in the article/**Supplementary Material**. Further inquiries can be directed to the corresponding authors.

## Author contributions

XZ, XC, and WJ planned and designed the research. SJ, BL, ZL, XZ, and TL participated in photosynthesis and chlorophyll fluorescence measurement experiments. XC, WJ, and EH were responsible for the analysis of photosynthetic fluorescence data. XX and XS provided advice on photosynthetic fluorescence data analysis. WJ and EH were involved in manuscript editing. XC and XZ participated in the revision of the manuscript. All authors read and approved the final manuscript.

## Funding

This research was funded by the National Key R&D Program of China, Grant Number 2019YFD1001500; the Basic Public Welfare Research Project in Zhejiang Province, Grant Number LGN22C160006; and the Talent Project of Jiyang College of Zhejiang A&F University, Grant Number RQ2020B04/RQ1911B05.

## Conflict of interest

The authors declare that the research was conducted in the absence of any commercial or financial relationships that could be construed as a potential conflict of interest.

## Publisher’s note

All claims expressed in this article are solely those of the authors and do not necessarily represent those of their affiliated organizations, or those of the publisher, the editors and the reviewers. Any product that may be evaluated in this article, or claim that may be made by its manufacturer, is not guaranteed or endorsed by the publisher.

## References

[B1] Bita C. E. Gerats T. (2013). Plant tolerance to high temperature in a changing environment: scientific fundamentals and production of heat stress-tolerant crops. Front. Plant Sci. 4. doi: 10.3389/fpls.2013.00273 PMC3728475 23914193

[B2] Blankenship R. E. (2014). “The basic principles of photosynthetic energy storage,” in Molecular mechanisms of photosynthesis, 2nd Edition (Chichester: John Wiley & Sons), 312.

[B3] Cai J. G. Wei M. Q. Zhang Y. We Y. (2017). Effects of shading on photosynthetic characteristics and chlorophyll fluorescence parameters in leaves of hydrangea macrophylla. Chin. J. Plant Ecology. 5), 570–576. doi: 10.17521/cjpe.2016.0245

[B506] Chalanika H. De Silva C. Asaeda T. (2017). Effects of heat stress on growth, photosynthetic pigments, oxidative damage and competitive capacity of three submerged macrophytes. J Plant Interact 12, 228–236. doi: 10.1080/17429145.2017.1322153

[B4] Chen H. X. Li W. J. An S. Z. Gao H. Y. (2004). Characterization of PSII photochemistry and thermostability in salt-treated *Rumex* leaves. J. Plant Physiol. 161, 257–264. doi: 10.1078/0176-1617-01231 15077623

[B5] Chen W. R. Zheng J. S. Li Y. Q. Guo W. D. (2012). Effects of high temperature on photosynthesis, chlorophyll fuorescence, chloroplast ultrastructure, and antioxidant activities in fngered citron. Russ J. Plant Physiol. 59, 732–740. doi: 10.1134/s1021443712060040

[B6] Derks A. Schaven K. Bruce D. (2015). Diverse mechanisms for photoprotection in photosynthesis. dynamic regulation of photosystem II excitation in response to rapid environmental change. Biochim. Biophys. Acta Bioenerg. 1847 (4-5), 468–485. doi: 10.1016/j.bbabio.2015.02.008 25687894

[B7] Du G. D. Lv D. G. Zhao L. Wang S. S. Cai Q. (2011). Effects of high temperature on leaf photosynthetic characteristics and photosystemII photochemical activity of kernelused apricot. Chin. J. Appl. Ecology. 22 (3), 701–706. 21657027

[B8] Du K. Wu W. Q. Liao T. Yang J. Kang X. Y. (2022). Transcriptome analysis uncovering regulatory networks and hub genes of populus photosynthesis and chlorophyll content. Genomics. 114 (4), 110385. doi: 10.1016/j.ygeno.2022.110385 35569730

[B9] Fahad S. Ihsan M. Z. Khaliq A. Daur I. Saud S. Alzamanan S.. (2018). Consequences of high temperature under changing climate optima for rice pollen characteristics-concepts and perspectives. Arch. Agron. Soil Sci. 64 (11), 1473–1488. doi: 10.1093/aob/mci071

[B10] Gao G. L. Feng Q. Zhang X. Y. Si J. H. Yu T. F. (2018). An overview of stomatal and non-stomatal limitations to photosynthesis of plants. Arid Zone Res. 35 (4), 929–937.

[B11] Gross Y. Kigel J. (1994). Differential sensitivity to high temperature of stages in the reproductive development of common bean (*Phaseolus vulgaris* l.). Field Crops Res. 36 (3), 201–212. doi: 10.1016/0378-4290(94)90112-0

[B13] Guo Y. Lu Y. Goltsev V. Strasser R. J. Kalaji H. M. Wang H.. (2020). Comparative effect of tenuazonic acid, diuron, bentazone, dibromothymoquinone and methyl viologen on the kinetics of chl a fluorescence rise OJIP and the MR820 signal. Plant Physiol. Biochem 156, 39–48. doi: 10.1016/j.plaphy.2020.08.044 32906020

[B12] Guo L. P. Wang Y. J. Jaime A. (2019). Teixeira da Silva, yongming fan, xiaonan yu. transcriptome and chemical analysis reveal putative genes involved in flower color change in *Paeonia* ‘Coral sunset’. Plant Physiol. Biochem. 138, 130–139. doi: 10.1016/j.plaphy.2019.02.025 30870763

[B14] Gu Z. Y. Zhu J. Hao Q. Yuan Y. W. Duan Y. W. Men S. Q.. (2019). (2019). a novel R2R3-MYB transcription factor contributes to petal blotch formation by regulating organ-specific expression of PsCHS in tree peony (*Paeonia suffruticosa*). Plant Cell Physiol. 60 (3), 599–611. doi: 10.1093/pcp/pcy232 30496505

[B15] Hao Z. J. Zhou C. H. Liu D. Wei M. R. Tao J. (2017). Effects of high temperature stress on photosynthesi, chlorophyll fluorescence and ultrastructure of herbaceous Peony(*Paeonia lactiflora* pall.). Mol. Plant Breed. 6, 2359–2367.

[B16] Hemantaranjan A. Bhanu A. N. Singh M. N. Yadav D. K. Patel P. K. Singh R.. (2014). Heat stress responses and thermotolerance. Adv. Plants Agri Res. 3, 1–10. doi: 10.15406/apar.2014.01.00012

[B17] Jiang D. Dai T. Jing Q. Cao W. Zhou Q. Zhao H.. (2004). Effects of long-term fertilization on leaf photosynthetic characteristics and grain yield in winter wheat. Photosynthetica 42, 439–446. doi: 10.1023/b:phot.0000046164.77410

[B18] Kalaji H. M. Bąba W. Gediga K. Goltsev V. Samborska I. A. Cetner M. D.. (2018). Chlorophyll fluorescence as a tool for nutrient status identification in rapeseed plants. Photosynth. Res. 136, 329–343. doi: 10.1007/s11120-017-0467-7 29185137 PMC5937862

[B19] Kalaji H. M. Jajoo A. Oukarroum A. Brestic M. Zivcak M. Samborska A. I.. (2014a). “The use of chlorophyll fluorescence kinetics analysis to study the performance of photosynthetic machinery in plants,” in Emerging technologies and management of crop stress tolerance. Eds. Ahmad P. Rasool S. (San Diego: Academic Press), 347–384.

[B20] Kalaji H. M. Schansker G. Ladle R. J. Goltsev V. Bosa K. Allakhverdiev I. S.. (2014b). Frequently asked questions about *in vivo* chlorophyll fluorescence: practical issues. Photosynth. Res. 122, 121–158. doi: 10.1007/s11120-014-0024-6 25119687 PMC4210649

[B21] Kharshiing E. Sinha S. P. (2016). Deficiency in phytochrome a alters photosynthetic activity, leaf starch metabolism and shoot biomass production in tomato. J. Photoch Photobio B 165, 157–162. doi: 10.1016/j.jphotobiol.2016.10.026 27794221

[B22] Krause G. H. Weis E. (1991). Chlorophyll fluorescence and photosynthesis: the basics. Annu. Rev. Plant Physiol. Plant Mol. Biol. 42, 313–349. doi: 10.1146/annurev.pp.42.060191.001525

[B23] Lesk C. Rowhani P. Ramankutty N. (2016). Influence of extreme weather disasters on global crop production. Nat. 529 (7584), 84–87. doi: 10.1038/nature16467 26738594

[B28] Liu J. J. (2019). Effect of high temperature and drought stress on PSII function and light distribution on peony leaves with different resistance. Northern Horticulture 11), 72–79.

[B26] Liu C. Y. Chen D. Y. Gai S. P. Zhang Y. X. Zheng G. S. (2012). Effects of high-and low temperature stress on the leaf PSII functions and physiological characteristics of tree peony (*Paeonia suffruticosa* cv. ‘Roufurong’). Chinese J. Appl. Ecol. 23 (1), 133–139. 22489490

[B27] Liu H. C. Zhu K. Y. Tan C. Zhang J. Q. Zhou J. H. Jin L.. (2019). Identification and characterization of *PsDREB2* promoter involved in tissue specific expression and abiotic stress response from paeonia suffruticosa. Peer J. 7, e7052. doi: 10.7717/peerj.7052 31223528 PMC6571008

[B24] Li J. Zhang X. Zhao X. (2011). Tree peony in China (Beijing, China: Encyclopedia of China Publishing House), 20–22.

[B25] Li M. Zhao G. H. Liu J. Liang X. P. Zhang M. Zhou G. C.. (2021). Optimization of ultrasound-assisted extraction of peony seed oil with response surface methodology and analysis of fatty acid. Agric. Res. 10 (4), 543–555. doi: 10.1007/s40003-021-00554-y

[B30] Lu T. Meng Z. J. Zhang G. X. Qi M. F. Sun Z. P. Liu Y. F.. (2017). Sub-High temperature and high light intensity induced irreversible inhibition on photosynthesis system of tomato plant (*Solanum lycopersicum* l.). Front. Plant Sci. 8, 365. doi: 10.3389/fpls.2017.00365 28360922 PMC5352666

[B29] Luo J. Han J. R. Wang Y. Ke L. Yang M. Fei Y. J. (2011). Response of heat stress on the physiological biochemistry of *Paeonia suffruticosa* . J. Yangtze Univ. (Nat Sci. Edit). 8 (2), 223–228.

[B31] Mathur S. Jajoo A. Mehta P. Bharti S. (2011). Analysis of elevated temperature-induced inhibition of photosystem II using chlorophyll a fluorescence induction kinetics in wheat leaves (*Triticum aestivum*). Plant Biol. 13, 1–6. doi: 10.1111/j.1438-8677.2009.00319.x 21143718

[B509] Mathur S. Mehta P. Anjana J. (2013). Effects of dual stress (high salt and high temperature) on the photochemical efficiency of wheat leaves (Triticum aestivum). Physiol. Mol. Biol. Plants 19 (2), 179–188. 24431485 10.1007/s12298-012-0151-5 PMC3656182

[B32] Maxwell K. Johanson G. N. (2000). Chlorophyll fluorescence - a practical guide. J. Exp. Bot. 51, 659–668. doi: 10.1093/jxb/51.345.659 10938857

[B33] Mihaljević I. Lepeduš H. Šimić D. Viljevac Vuletić M. Tomaš V. Vuković D.. (2020). Photochemical efficiency of photosystem II in two apple cultivars affected by elevated temperature and excess light *in vivo* . S. Afr. J. Bot. 130, 316–326. doi: 10.1016/j.sajb.2020.01.017

[B34] Miner G. L. Bauerle W. L. Baldocchi D. D. (2017). Estimating the sensitivity of stomatal conductance to photosynthesis: A review. Plant Cell Environ. 40 (7), 1214–1218. doi: 10.1111/pce.12871 27925232

[B35] Mittler R. Finka A. Goloubinoff P. (2012). How do plants feel the heat? Trends Biochem. Sci. 37 (3), 118–125. doi: 10.1016/j.tibs.2011.11.007 22236506

[B36] Morita S. Yonemaru J. Takanashi J. (2005). Grain growth and endosperm cell size under high night temperatures in rice (*Oryza sativa* l.). Ann. Bot. 95 (4), 695–701. doi: 10.1093/aob/mci071 15655104 PMC4246861

[B37] Olsovska K. Brestic M. (2001). Function of hydraulic and chemical water stress signalization in evaluation of drought resistance of juvenile plants. J. Cent. Eur. Agric. 2 (34), 157–164.

[B505] Öquist G. Chow W. S. Anderson J. M. (1992). Photoinhibition of photosynthesis represents a mechanism for the long-term regulation of photosystem II. Planta 186, 450–460. doi: 10.1007/bf00195327 24186743

[B38] Pavlović I. Mlinarić S. Tarkowská D. Oklestkova J. Novák O. Lepeduš H.. (2019). Early brassica crops responses to salinity stress: A comparative analysis between Chinese cabbage, white cabbage, and kale. Front. Plant Sci. 10. doi: 10.3389/fpls.2019.00450 PMC6470637 31031786

[B39] Ren Z. B. Chen F. Z. Shu C. Q. Li X. H. Liu K. H. Ji X. M. (2018). Effects of exogenous 2, 4-epibrassinolide on heat resistance of peony. J. Jianghan Univ.(Nat. Sci. Ed.). 46 (5), 446–453.

[B40] Sarwar M. Saleem M. F. Ullah N. Ali S. Rizwan M. Shahid M. R.. (2019). Role of mineral nutrition in alleviation of heat stress in cotton plants grown in glasshouse and field conditions. Sci. Rep. 9 (1), 13022. doi: 10.1038/s41598-019-49404-6 31506449 PMC6737086

[B503] Scalet M. Federice R. Guido M. C. (1995). Peroxidase activity and polyamine changes in response to ozone and simulated acid rain in Aleppo pine needles. Environ. Exp. Bot. 35 (3), 417–425. doi: 10.1016/0098-8472(95)00001-3

[B501] Srivalli B. Vishanathan C. Renu K. C. (2003). Antioxidant defense in response to abiotic stresses in plants. Journal of Plant Biology 30, 121–139.

[B41] Srivastava A. Guiss´e B. Greppin H. Strasser R. J. (1997). Regulation of antenna structure and electron transport in photosystem II of pisum sativum under elevated temperature probed by the fast polyphasic chlorophyll a fluorescence transient: OKJIP. Biochim. Biophys. Acta 1320, 95–106. doi: 10.1016/s0005-2728(97)00017-0

[B507] Stefanov D. Petkova V. Denev I. D. (2011). Screening for heat tolerance in commonbean (Phaseolus vulgaris L) lines and cultivars using JIP test. Sci. Hortic 128, 1–6. doi: 10.1016/j.scienta.2010.12.003

[B508] Stirbet A. D. Strasser R. J. (1996). Numerical simulation of the in vivo fluorescence in plants. Math. Comput. Simul. 42, 245–53. doi: 10.1016/0378-4754(95)00114-x

[B42] Stirbet A. (2013). Excitonic connectivity between photosystem II units: What is it, and how to measure it? Photosynth. Res. 116, 189–214. doi: 10.1007/s11120-013-9863-9 23794168

[B47] Strasser R. J. Appenroth,. K. J. StÖckel J. Srivastava A. (2001). Multiple effect of chromate on the photosynthetic apparatus of spirorlela polyrhiza probed by OJIP chlorophyll a fluorescence measurements. Environ. pollut. 115 (1), 49–64. doi: 10.1016/s0269-7491(01)00091-4 11586773

[B44] Strasser R. J. Govindjee (1991). “The fo and the O-J-I-P fluorescence rise in higher plants and algae,” in Regulation of chloroplast biogenesis. Ed. Argyroudi-Akoyunoglou J. H. (New York: Plenum Press), 423–436.

[B43] Strasser B. J. Strasser R. J. (1995). “Measuring fast fluorescence transients to address environmental questions: the JIP-test,” in Photosynthesis: from light to biosphere. Ed. Mathis P. (Netherland: Kluwer Academic Publishers Press), 977–980.

[B45] Strasser R. J. Tsimilli-Michael M. (2001). Stress in plants, from daily rhythm to global changes, detected and quantified by the JIP-test. Chimie Nouvelle (SRC). 75, 3321–3326.

[B46] Strasser R. J. Tsimilli-Michael M. Srivastava A. (2004). Analysis of the chlorophyll a fluorescence transient. Chlorophyll Fluorescence 321–362. doi: 10.1007/978-1-4020-3218-9_12

[B48] Sun X. L. Li W. G. Li J. Zu Y. G. Zhao X. H. (2017). Inclusion complex of peony (*Paeonia suffruticosa* andr.) seed oil with b-cyclodextrin: preparation, characterisation and bioavailability enhancement. Int. J. Food Sci. Technol. 52 (11), 2352–2361. doi: 10.1111/ijfs.13519

[B49] Tao M. Z. Feng X. P. He Y. Zhang J. N. Bai X. L. Yang G. F.. (2022). Monitoring of transgenic maize seedlings phenotyping exhibiting glyphosate tolerance. Biorxiv. Org. doi: 10.1101/2022.03.21.485126

[B50] Tiwari Y. K. Yadav S. K. (2019). High temperature stress tolerance in maize (Zea mays l.): Physiological and molecular mechanisms. J. Plant Biol. 62 (2), 93–102. doi: 10.1007/s12374-018-0350-x

[B51] Tsimilli-Michael M. (2019). Revisiting JIP-test: an educative review on concepts, assumptions, approximations, definitions and terminology. Photosynthetica 57 (SI), 90–107. doi: 10.32615/ps.2019.150

[B52] Van Heerden P. D. R. Strasser R. J. Kruger G. H. J. (2004). Reduction of dark chilling stress in N2-fixing soybean by nitrate as indicated by chlorophyll a fluorescence kinetics. Physiol. Plant 121 (2), 239–249. doi: 10.1111/j.0031-9317.2004.0312.x 15153191

[B53] Viljevac Vuletić M. Španić V. (2019). Characterization of photosynthetic performance during natural leaf senescence in winter wheat: Multivariate analysis as a tool for phenotypic characterization. Photosynthetica 57 (SI), 116–128. doi: 10.32615/ps.2019.162

[B54] Wang Y. Z. Jiang H. F. Fu L. M. Zhang X. J. (2012). Summer management points of greenhouse potted peony. Rural Sci. Technol. 10, 56.

[B502] Wang L.-J. Li S.-H. (2006). Thermotolerance and related antioxidant enzyme activities induced by heat acclimation and salicylic acid in grape (*Vitis vinifera* L.) leaves. Plant Growth Regulation 48, 137–144.

[B55] Wen S. S. Cheng F. Y. Zhong Y. Wang X. Li L. Z. Zhang Y. X.. (2016). Efficient protocols for the micropropagation of tree peony (*Paeonia suffruticosa* ‘Jin pao hong’, *P. suffruticosa* ‘Wu long peng sheng’ and p. × *lemoinei*’High noon’) and application of arbuscular mycorrhizal fungi to improve plantlet establishment. Sci. Hortic. 201, 10–17. doi: 10.1016/j.scienta.2016.01.022

[B56] Wu S. Jin X. L. Jin M. H. Sun L. X. Chen R. (2018). Effects of exogenous abscisic acid on heat tolerance in tree peony seedlings under high temperature stress. advances in ornamental. Horticulture China 346–352.

[B58] Xu D. Q. (2013). Photosynthesis (Beijing, China: Science Press).

[B59] Xue J. Q. Li T. T. Wang S. L. Xue Y. Q. Hu F. R. Zhang X. X. (2018). Elucidation of the mechanism of reflowering in tree peony (*Paeonia suffruticosa*) ‘Zi luo lan’ by defoliation and gibberellic acid application. Plant Physiol. Biochem. 132, 571–578. doi: 10.1016/j.plaphy.2018.10.004 30326436

[B57] Xu C. Wang M. C. Yang Z. Q. Han W. Zheng S. H. (2021). Effects of high temperature on photosynthetic physiological characteristics of strawberry seedlings in greenhouse and construction of stress level. Chinese J. Appl. Ecol. 32 (1), 231–240 10.13287/j.1001-9332.202101.028 33477231

[B60] Yu S. A. (2019). The impact of climate change on crop production: an empirical study in Zhejiang, China. Dissertation: China National Knowledge Infrastructure database. Zhejiang: Zhejiang University.

[B61] Zhang L. X. Chang Q. S. Hou X. G. Wang J. Z. Chen S. D. Zhang Q. M.. (2022). The effect of High−Temperature stress on the physiological indexes, chloroplast ultrastructure, and photosystems of two herbaceous peony cultivars. J. Plant Growth Regul. doi: 10.1007/s00344-022-10647-9

[B63] Zhang Y. Z. Cheng Y. W. Ya H. Y. Han J. M. Zheng L. (2015). Identification of heat shock proteins *via* transcriptome profiling of tree peony leaf exposed to high temperature. Genet. Mol. Res. 14 (3), 8431–8442. doi: 10.4238/2015.July.28.10 26345770

[B62] Zhang S. Q. Shan L. (2002). Research progress on water use efficiency of plant. Agric. Reseach In Arid Areas 20 (4), 1–5.

[B64] Zhou F. F. Wang Z. Shi L. Niu J. J. Shang W. He D.. (2016). Effects of different medium composition and exogenous hormones on browning of tree peony (*Paeonia suffruticosa* andr.) callus in tissue culture. Flower Res. J. 24, 96–102. doi: 10.11623/frj.2016.24.2.03

[B65] Zhu S. H. Ma J. Hao L. H. Zhang L. L. (2021). Identification and analysis of heat-resistant differential protein in leaves of *Paeonia suffruticosa* . Mol. Plant Breed. 19 (2), 419–431.

